# Supplementary material for: Genetics and evidence for balancing selection of a sex-linked colour polymorphism in a songbird
Source: Nat Commun. 2019 Apr 23;10:1852. doi: 10.1038/s41467-019-09806-6 (PMC6478913; doi:10.1038/s41467-019-09806-6)
Supplement: Supplementary file 3 — Reporting Summary [file 41467_2019_9806_MOESM3_ESM.pdf]

nature research

Corresponding author(s): Kang-Wook Kim
Kai Zeng
Terry Burke

Last updated by author(s): Mar 15, 2019

Reporting Summary

Nature Research wishes to improve the reproducibility of the work that we publish. This form provides structure for consistency and transparency in reporting. For further information on Nature Research policies, see Authors & Referees and the Editorial Policy Checklist.

Statistics

For all statistical analyses, confirm that the following items are present in the figure legend, table legend, main text, or Methods section.

n/a	Confirmed	
☐	☒	The exact sample size (*n*) for each experimental group/condition, given as a discrete number and unit of measurement
☒	☐	A statement on whether measurements were taken from distinct samples or whether the same sample was measured repeatedly
☒	☐	The statistical test(s) used AND whether they are one- or two-sided<br>*Only common tests should be described solely by name; describe more complex techniques in the Methods section.*
☒	☐	A description of all covariates tested
☐	☒	A description of any assumptions or corrections, such as tests of normality and adjustment for multiple comparisons
☐	☒	A full description of the statistical parameters including central tendency (e.g. means) or other basic estimates (e.g. regression coefficient) AND variation (e.g. standard deviation) or associated estimates of uncertainty (e.g. confidence intervals)
☒	☐	For null hypothesis testing, the test statistic (e.g. *F*, *t*, *r*) with confidence intervals, effect sizes, degrees of freedom and *P* value noted<br>*Give P values as exact values whenever suitable.*
☐	☒	For Bayesian analysis, information on the choice of priors and Markov chain Monte Carlo settings
☒	☐	For hierarchical and complex designs, identification of the appropriate level for tests and full reporting of outcomes
☒	☐	Estimates of effect sizes (e.g. Cohen's *d*, Pearson's *r*), indicating how they were calculated

Our web collection on statistics for biologists contains articles on many of the points above.

Software and code

Policy information about availability of computer code

Data collection	N/A
Data analysis	NGS Sequence analysis: process_radtags and clone_filter utility programs from STACKS v2.1, FASTQC v0.10.0, SeqMan NGen 4.0 (DNASTAR), SAMTOOLS v1.2, VCFtools v0.1.12, PLINK v1.9, GATK v.3.4.0, Bowtie 2 v2.2.0, TagCle v0.80 (a custom Perl script available upon request from K.-W.K.).<br>Sequence analysis: GENEMAPPER v 3.7 (Applied Biosystems), CodonCode Aligner v2.0.6 (CodonCode Corporation), UGENE v1.12.3, BioEdit v7.1.11.<br>Transcriptome analysis: Expression Console v1.3 (Affymetrix), Transcriptome Analysis Console v2.0 (Affymetrix), Cytoscape plugin ClueGO v2.3.5.<br>Population genetic/phylogenetic analysis: HardyWeinberg v1.6.1, Haploview v4.2, DnaSP v5, CERVUS v3.0.3, STRUCTURE v2.3.4, fineRADstructure v0.3.2, phangorn v2.3.1, pegas v0.10.<br>Coalescent simulation: Custom scripts for a modified version of the HKA test, the method of Zeng and Charlesworth (2009, Genetics), SelSim, mbs, and custom R and Python scripts to calculate site-by-site Fst values, Dxy and π across the candidate region using sliding-window analyses.<br>R packages for data visualization: ggplot2 v2.2.1, ggtree v1.4.20, LDheatmap v.0.99.2, rtracklayer v.1.30.4, Gviz v.1.14.7.<br>All data analysis was conducted using R v3.2.5, unless stated otherwise.

For manuscripts utilizing custom algorithms or software that are central to the research but not yet described in published literature, software must be made available to editors/reviewers. We strongly encourage code deposition in a community repository (e.g. GitHub). See the Nature Research guidelines for submitting code & software for further information.

Data

Policy information about availability of data

All manuscripts must include a data availability statement. This statement should provide the following information, where applicable:

- Accession codes, unique identifiers, or web links for publicly available datasets
- A list of figures that have associated raw data
- A description of any restrictions on data availability

The data that support the findings of this study are available from the corresponding authors on request. PLINK files for RADSeq analysis and sequence alignments of de novo sequencing for the Red locus underlying Figs 1a and 4b, Supplementary Figs 1, 2, 6 and 8-19 and Supplementary Tables 9 and 11-13 are available from the Dryad Digital Repository [https://doi.org/10.5061/dryad.9nk3757]. Raw Illumina reads are available at NCBI SRA (SAMN10751906-SAMN10751929) under BioProject PRJNA515277 [https://www.ncbi.nlm.nih.gov/bioproject/PRJNA515277]. Microarray data are available on the Gene Expression Omnibus (GEO) under accession number GSE125295 [https://www.ncbi.nlm.nih.gov/geo/query/acc.cgi?acc=GSE125295]. A reporting summary for this article is available as a Supplementary Information file.

Field-specific reporting

Please select the one below that is the best fit for your research. If you are not sure, read the appropriate sections before making your selection.

☒ Life sciences ☐ Behavioural & social sciences ☐ Ecological, evolutionary & environmental sciences

For a reference copy of the document with all sections, see nature.com/documents/nr-reporting-summary-flat.pdf

Life sciences study design

All studies must disclose on these points even when the disclosure is negative.

Sample size

No power analysis was performed prior to the study.

Data exclusions

In the first batch, DNA fragment libraries for 20 individuals (nblack_male = 10, nred_female = 8 and nred_male = 2) were constructed.
In the second batch, a pooled library including 12 additional black males from the same population was constructed.
In an Fst outlier analysis (Supplementary Fig. 1), we included 32 individuals (nblack_male = 22, nred_female = 8 and nred_male = 2) whose genotypes at the Red locus were either homozygous or hemizygous, except for the two heterozygous red males.
To increase the chance of detecting the location of the Red locus in an Fst outlier analysis for the samples from American captive population (Supplementary Fig. 1), we only included data from 17 individuals whose genotypes were known to be homozygous or hemizygous for alternative alleles at the Red locus based on the pedigree (black: nblack male = 4, nblack female = 3; red: nred female = 5 and nyellow female = 5).
To confirm the association with plumage colour, 161 birds were genotyped for alternative two-base pair variants.
Genomic DNA from 16 females (nblack = 8, nred = 8) was Sanger sequenced for 26 loci in LD analysis.
The coding sequences of follistatin in 16 females were aligned, along with the homologous assembled zebra finch sequence, to examine the differences between species and morphs.
To test for an inversion across the Red locus, 19 contiguous, overlapping fragments were amplified in 12 females (nblack = 6, nred = 6) from the wild population.
We obtained the gene expression profiles of 6 birds (nblack_male = 3, nred_male = 2, nred_female = 1).
Two individuals had significantly high levels of missing data and were excluded from calculation of the coancestry matrix.
In a STRUCTURE analysis using microsatellite markers we genotyped 161 birds (nblack = 126, and nred = 35).
Blood samples of 14 species in the family Estrildidae including red-faced (E. pealii, E. psittacae) or blue-faced (E. tricolor and E. trichroa) Erythrura species were used in gene genealogy and relative rate test.

Replication

N/A

Randomization

A subset of samples was randomly selected and used for RADSeq and subsequent Sanger and MiSeq sequencing and genotyping.

Blinding

Throughout the study, traits were measured blind to treatment and to values of other traits.

Reporting for specific materials, systems and methods

We require information from authors about some types of materials, experimental systems and methods used in many studies. Here, indicate whether each material, system or method listed is relevant to your study. If you are not sure if a list item applies to your research, read the appropriate section before selecting a response.

Materials & experimental systems

n/a	Involved in the study
☒	☐ Antibodies
☒	☐ Eukaryotic cell lines
☒	☐ Palaeontology
☐	☒ Animals and other organisms
☒	☐ Human research participants
☒	☐ Clinical data

Methods

n/a	Involved in the study
☒	☐ ChIP-seq
☒	☐ Flow cytometry
☒	☐ MRI-based neuroimaging

Animals and other organisms

Policy information about studies involving animals; ARRIVE guidelines recommended for reporting animal research

Laboratory animals	N/A
Wild animals	Blood samples were collected from 161 Gouldian finches (male: nblack = 62, nred = 27; female: nblack = 64, nred = 8) in 2008 from a single wild population in Western Australia (within a 50-km radius of Wyndham in East Kimberley). Birds were either caught in mist nets at waterholes or at their nests, and released after blood sampling The population size was estimated to be around 300 adults.
Field-collected samples	N/A
Ethics oversight	Blood sampling of birds was conducted under the approval of the Animal Ethics Committees at the University of New South Wales and Macquarie University (ARAs 2007/037 & 2007/038).

Note that full information on the approval of the study protocol must also be provided in the manuscript.
