# Supplementary material for: Genetics and evidence for balancing selection of a sex-linked colour polymorphism in a songbird
Source: Nat Commun. 2019 Apr 23;10:1852. doi: 10.1038/s41467-019-09806-6 (PMC6478913; doi:10.1038/s41467-019-09806-6)
Supplement: Supplementary file 4 — Description of Additional Supplementary Files [file 41467_2019_9806_MOESM4_ESM.pdf]

**Description of Additional Supplementary Files**

File Name: Supplementary Data 1
Description: Summary of transcriptome analysis for the regenerating feathers.
